# TCR-transgenic T cells and YB-1-based oncolytic virotherapy improve survival in a preclinical Ewing sarcoma xenograft mouse model

**DOI:** 10.3389/fimmu.2024.1330868

**Published:** 2024-01-22

**Authors:** Sebastian J. Schober, Melanie Thiede, Hendrik Gassmann, Anna Josefine von Ofen, Pia Knoch, Jennifer Eck, Carolin Prexler, Corazon Kordass-Wally, Julia Hauer, Stefan Burdach, Per Sonne Holm, Uwe Thiel

**Affiliations:** ^1^Department of Pediatrics, Children’s Cancer Research Center, Kinderklinik München Schwabing, TUM School of Medicine and Health, Technical University of Munich, Munich, Germany; ^2^Institute of Pathology, Klinikum rechts der Isar, TUM School of Medicine and Health, Technical University of Munich, Munich, Germany; ^3^Department of Urology, Klinikum rechts der Isar, TUM School of Medicine and Health, Technical University of Munich, Munich, Germany; ^4^Department of Oral and Maxillofacial Surgery, Medical University Innsbruck, Innsbruck, Austria

**Keywords:** pediatric sarcoma, Ewing sarcoma, oncolytic virotherapy, TCR-transgenic T cells, combination immunotherapy

## Abstract

**Background:**

Ewing sarcoma (EwS) is an aggressive and highly metastatic bone and soft tissue tumor in pediatric patients and young adults. Cure rates are low when patients present with metastatic or relapsed disease. Therefore, innovative therapy approaches are urgently needed. Cellular- and oncolytic virus-based immunotherapies are on the rise for solid cancers.

**Methods:**

Here, we assess the combination of EwS tumor-associated antigen CHM1^319^-specific TCR-transgenic CD8^+^ T cells and the YB-1-driven (i.e. E1A13S-deleted) oncolytic adenovirus XVir-N-31 *in vitro* and in a xenograft mouse model for antitumor activity and immunostimulatory properties.

**Results:**

*In vitro* both approaches specifically kill EwS cell lines in a synergistic manner over controls. This effect was confirmed *in vivo*, with increased survival using the combination therapy. Further *in vitro* analyses of immunogenic cell death and antigen presentation confirmed immunostimulatory properties of virus-infected EwS tumor cells. As dendritic cell maturation was also increased by XVir-N-31, we observed superior proliferation of CHM1^319^-specific TCR-transgenic CD8^+^ T cells only in virus-tested conditions, emphasizing the superior immune-activating potential of XVir-N-31.

**Conclusion:**

Our data prove synergistic antitumor effects *in vitro* and superior tumor control in a preclinical xenograft setting. Combination strategies of EwS-redirected T cells and YB-1-driven virotherapy are a highly promising immunotherapeutic approach for EwS and warrant further evaluation in a clinical setting.

## 1 Introduction

Immunotherapeutic targeting of Ewing sarcoma (EwS) has proven to be very complex. Both systemic immunosuppression as well as a T cell-hostile tumor microenvironment (TME) have to be overcome for successful therapy (1–3). In this regard, the effectiveness of targeting immune checkpoint molecules (ICB, immune checkpoint blockade), such as PD-1 or CTLA-4, has not shown to be a promising therapy option in its present form as mono/dual-ICB (4, 5). A possible explanation for current failure of ICB is the low mutational, hence neoantigen burden, resulting in a relatively narrow T cell receptor (TCR) repertoire and scarce T cell infiltration of tumors, as compared to many adult cancers (6, 7). Apart from this, a TME with immunosuppressive soluble and cellular factors, such as tumor-associated macrophages with tumorigenic and T cell inhibiting features, has to be addressed, especially as factors like abundant M2-macrophage signatures correlate with poor survival (3, 8). EwS are characterized by specific oncogenic fusion products, which could not have been targeted by drugs directly until now (9). Therefore, targeted molecular therapy only can be applied in an individualized setting against actionable targets other than the specific oncogenic product (10) and all relapsed/therapy refractory patients are still dependent on conventional radio-/chemotherapies, with current survival rates not exceeding 10-30% (9, 11, 12).

Direct immunotherapeutic targeting of natural peptides derived from the oncogenic fusion protein EWS-FLI1 has not been successful due to low peptide/MHC binding affinity and consecutive weak T cell responses (13, 14). Hence, the focus of TCR-based adoptive T cell transfer in EwS has mainly been on addressing cancer/testis antigens and overexpressed tumor-associated antigens (15–17). In this context, our group identified and characterized several TCRs derived from the allorepertoire with antitumor activity *in vitro* and *in vivo* (18–21). The most promising candidate TCR directed against a nonameric peptide from chrondromodulin-1 (CHM1^319^, CHM1, CNMD), a direct downstream target of EWS-FLI1 mediating metastasis is applied in this work (22). Despite promising *in vivo* result, demonstrating the feasibility of T cell therapy in EwS, T cell-mediated tumor control in solid tumor has remained an exception for most pediatric entities until today (23). Novel combination strategies are urgently needed in order to break physical and immunosuppressive barriers before adoptively transferring TCR or CAR (chimeric antigen receptor) T cells.

One elegant and currently reborn approach is the application of oncolytic viruses (OVs) which allows tumor targeting in a more general way. Pediatric sarcomas express high levels of the multifunctional Y-box binding protein 1 (YB-1), which is associated with drug resistance and metastatic properties. Additionally, survival probabilities decrease with higher YB-1 levels and nuclear location (24, 25). The application of the YB-1-dependent oncolytic adenovirus XVir-N-31 for these patient population is obvious (26). Certainly, OV monotherapies are very promising but long-lasting tumor control are only observed in a minority of patients, hence current strategies aim at optimizing replicative capacity or inducing stronger antitumor immune activity (27–30). This direction of impact is reflected clinically by combination approaches primarily using ICB, which have already shown an impressive increase in response and survival rates (31, 32). Adaptive immune system responses after OV are described to be mainly directed against viral epitopes or even neoepitopes and the combination of OVs together with tumor-redirected T cells is not only thought to increase tumor cell killing but also to reduce immune-evasion (33–35).

We herein hypothesized that CHM1^319^-specific T cells can be successfully combined with XVir-N-31 to address EwS, resulting in synergistic antitumor and immunostimulatory effects.

## 2 Materials and methods

### 2.1 Cell lines

A673 and HEK293 were purchased from the American Type Culture Collection (ATCC). LCL, MHH-ES1, THP-1, SK-N-MC were obtained from the German Collection of Microorganisms and Cell Cultures (DSMZ). TC32 was a kind gift from Prof. Poul Sorensen (University of British Columbia, Canada), which was originally purchased from the Childhood Cancer Repository (CCR, Alex’s Lemonade Stand Foundation, Children’s Oncology Group, COG). SB-KMS-KS1 was established at the Children’s Cancer Research Center, Kinderklinik Schwabing (Technical University Munich, Germany). HLA types of utilized cell lines are given in **Table 1**. IL15-producing NSO cells were a kind gift from Prof. Stanley Riddell (University of Washington School of Medicine), and the packaging cell line (293Vec.) RD114 was kindly provided by Prof. Manuel Caruso (Centre de recherche de Québec, Université Laval). Healthy peripheral blood mononuclear cells (PBMC) were purchased from DRK-Blutspendedienst after informed consent and approval of local government regulatory authorities. Mycoplasma testing was performed regularly (e.g., before *in vivo* usage, MycoAlert Mycoplasma Detection Kit, Lonza) and cell lines were cultures in accordance with the supplier’s recommendation.

**Table 1:** HLA types of EwS cell lines used in this study.

Cell line	HLA class I (36)
*A673*	A*01:01, **02:01**; B*07:02, 07:02, C*07:02;07:02
*MHH-ES1*	A*01:01, 68:01; B*40:01, 49:01, C*01:02;07:01
*SB-KMS-KS1*	A*02 negative, further information not available (37)
*SK-N-MC*	A*01:01, 25:01; B*08:01, 08:01; C*07:01,07:01
*THP-1*	A*02:01, 02:01; B*15:11, 15:11; C*03:03, 03:03
*TC32*	A*02:01, further information not available (38)

Bold values can be used to study CHM1-TCR recognition/activity.

### 2.2 Retroviral TCR construct

Detailed information on the identification and characterization of the herein used CHM1^319^/HLA-A*02:01-restricted TCR as well as the synthesis of retroviral constructs can be found in previous publications (18, 37).

### 2.3 Isolation of PBMC

Healthy donor PBMC were isolated by density-gradient-centrifugation as described previously (39). The Ficoll-Paque (GE Healthcare) was used according to supplier’s recommendation with subsequent application of ACK Lysis buffer (Thermo Fisher Scientific) to deplete remaining red blood cells.

### 2.4 Generation and expansion of CHM1^319^/HLA-A*02:01-restricted TCR-transgenic T cells

Therapeutic TCR-transgenic T cells were generated as reported before (18, 37). Starting material were health donor PBMC (HLA-A*02 negative). Then CD8^+^ T cells were selected negatively by using Miltenyi Biotech equipment (CD8^+^ T cell Isolation Kit, magnets, and L-size columns). TCR-transduction of T cells was performed retrovirally (pMP71 vector). CHM1^319^-specific multimers were used to detect and purify transgenic cells. Using anti-PE microbead enrichment (Miltenyi Biotech), purities > 95% were achieved. Purified TCR-transgenic T cells were expanded with feeder cells in 25 mL T cell medium (AIM-V, containing 5% hAB serum, penicillin 100 U/mL, streptomycin 100 g/mL). Feeder cells were utilized as follows: 5 x 10^6^ irradiated LCL (100 Gray, Gy) and 2.5 x 10^7^ irradiated PBMC (30 Gy, five different healthy PBMC donors were pooled) per 25 mL. For cell irradiation a BioBeam8000 machine was used. T cell cytokines were supplemented every 2 – 3 days (100 U/mL recombinant human IL2 from Novartis and 2 ng/mL recombinant human IL15 from R&D Systems).

### 2.5 Adenoviral vectors and infection

AdWT was used as a control when indicated and was originally obtained from Prof. David Curiel (Washington University, St. Louis, Missouri). Furthermore, a E1-deleted Ad vector expressing GFP und the control of a CMV promotor with the additional RGD motif was used to study the infectivity of tumor cells and was provided by Prof. Per Sonne Holm and Klaus Mantwill (Technical University of Munich). The YB-1-dependent oncolytic adenovirus XVir-N-31, first described as Ad-Delo3-RGD, was also provided by Prof. Per Sonne Holm and Klaus Mantwill. Here, deletions within the virome include the CR3-region of E1A, E1B19k as well as the E3-region, complemented by alterations at the fiber knob to express an RGD-motif (26, 40). All adenoviral constructs were produced in HEK293 cells and purified via CsCl-gradient (two-step) centrifugation followed by size-exclusion chromatography with PD-10 desalting columns (GE Healthcare). Viral infection was performed in 6-, 24-well-plates or 10cm-dishes with given MOI as described before. Downstream processing after viral infection is described in more detail in the results section or in corresponding figure legends (29).

### 2.6 Determination of adenoviral IFU

**Figure 1:**
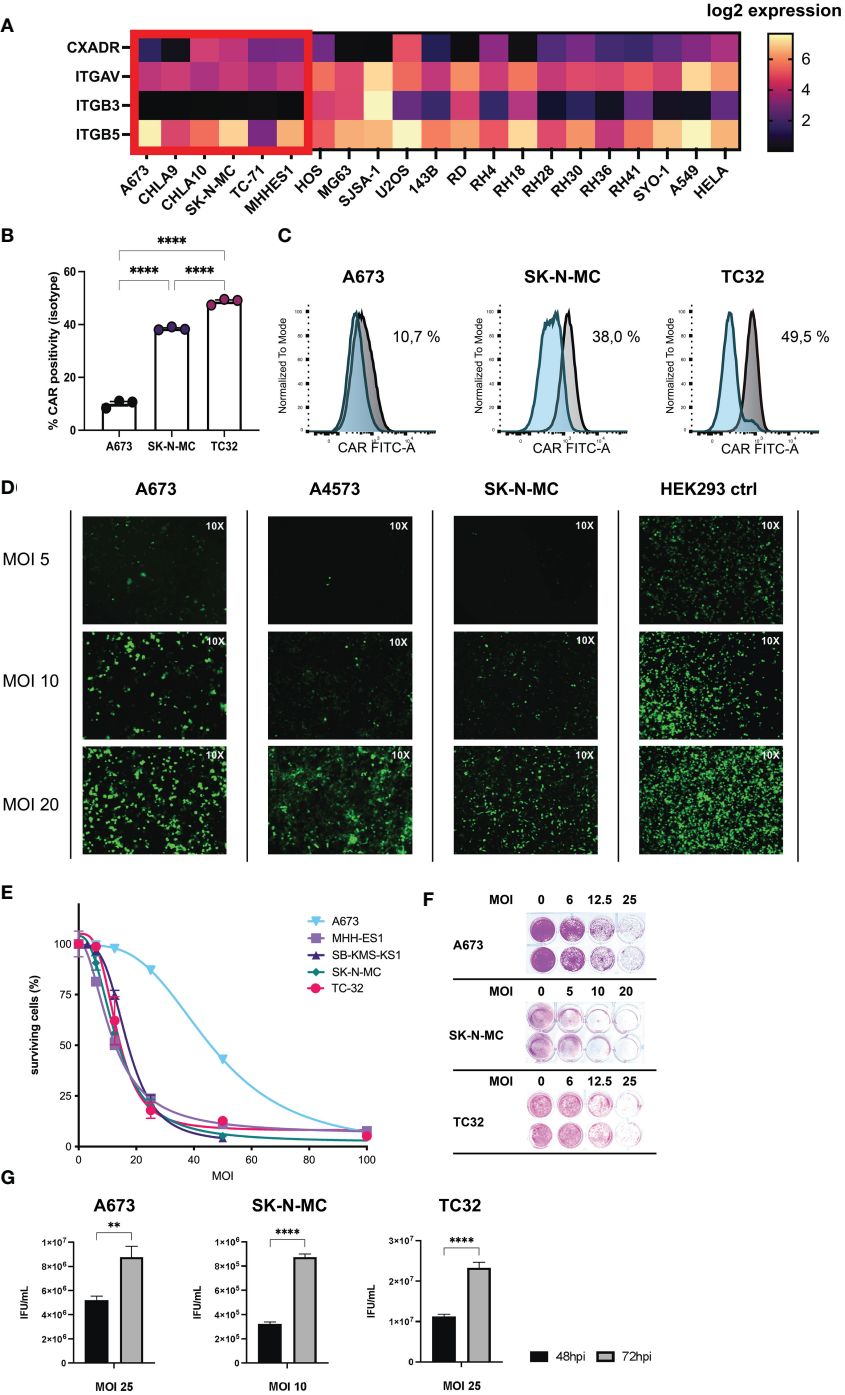
Ewing sarcoma (EwS) cell lines are susceptible to adenovirus (Ad) infection and XVir-N-31-infection induces tumor lysis and release of viral progeny. **(A)** Receptor expression in tumor cell lines associated with viral entry of XVir-N-31 (AdDelo3RGD). Log2 gene expression values of relevant receptors for interaction with Ad-RGD (CXADR, ITGAV, ITGB3, ITGB5), were extracted from DepMap Portal *Cell Line Selector* (Broad Institute: depmap.org) and visualized with the heatmap tool in Prism 9. EwS cell lines are marked in red; additional osteosarcoma and rhabdomyosarcoma cell lines as well as A549 and HELA are also shown for comparison. **(B, C)** FACS analysis of cognate Ad5 receptor (CAR, CXADR) surface expression on tumor cell lines (A673, SK-N-MC, TC32), examined with an anti-CAR-FITC antibody, and plotted as column bars of CAR-positive cells (minus isotype) in **(B)** and depicted as **(C)** histograms of the mean fluorescence intensity (normalized to mode) of CAR signal (*grey*) and isotype control (*blue*). **(D)** Multiplicity of infection (MOI)/dose-dependent increase of infectivity analyzed by immunofluorescence imagining (10x) of indicated EwS tumor cells and HEK293 control after virus infection with a GFP-expressing Ad-RGD. Photos were taken 48 hours post infection (hpi). **(E, F)** Cell survival assay of a selection of tumor cell lines after infection with XVir-N-31 at indicated MOI, analyzed 4-6 days post infection (dpi) with SRB staining and photometric extinction measurement. **(G)** Formation of viral progeny was assessed by hexon titer tests of EwS cell lysates 48hpi and 72hpi respective MOI and depicted as infectious units (IFU)/mL for 3 representative cell lines. Levels of significance are indicated as asterisks: * p < 0.05, ** p < 0.01, *** p < 0.005, **** p < 0.0001. Either unpaired student’s t-test or ordinary ANOVA with Tukey’s multiple comparison were used in Prism 9. Error bars show the SEM.

A hexon titer test was used to determine infectious viral units (IFU), as described before (29). In short, IFU were quantified with HEK293 cells and immunocytochemistry staining. Seeding of HEK293 and infection was performed simultaneously in 24-well-plates. After approximately 44 hours, cells were fixed with ice-cold methanol and stained with a primary anti-hexon (#AB1056, Merck Millipore, 1:1000) and a secondary horseradish peroxidas-conjugated antibody (#R0449, Dako, 1:1000). Liquid DAB + Substrate Chromogen System (Dako) was used for development. Hexon-expressing cells were counted (10 visual fields per well, 20x magnification) and viral titers were determined: titer (IFU/mL) = (average number of positive cells/field x fields/well)/[volume of diluted virus per well (mL) x dilution factor]. Error bars in **Figure 1G** show the SD.

### 2.7 Cell survival assays

Tumor cell killing by XVir-N-31 or therapeutic T cells was studied using either the sulforhodamine B (SRB) or the xCELLigence assay (Roche/ACEA). For the SRB assay, tumor cells were seeded overnight in 24-well-plates (20,000 - 30,000 cells per well; if different cell numbers were used, respective numbers are given in the figure legends). After viral infection cells were fixed 3-5 days afterwards with trichloroacetic acid (10%, at 4°C overnight), stained with SRB (Sigma-Aldrich, 0,5% SRB in 1% acetic acid for 30min) and washed afterwards before quantification of the cytopathic effect by dissolving SRB-stained cells in 10 mmol/L Tris buffer (pH 10). Extinction was determined at 510nm (Infinite M Nano, Tecan).

For determination of real-time cytotoxicity, the xCELLigence assay was used. Here, 10,000-20,000 tumor cells were plated in 96-well-E-plates in triplicates or quadruplicates. XVir-N-31 or therapeutic T cells were added at indicated time points and different MOI as well as E:T-ratios were used. Virus infection was performed at a cell index to 0.5 – 1.0 and addition of T cells at a cell index of 1.0 - 2.0.

### 2.8 Immunofluorescence microscopy

GFP-expressing tumor cells after viral infection (**Figure 1D**) were acquired and imaged at 10x magnification with an Axiovert 100 microscope using fluorescent light.

### 2.9 Generation of tumor conditioned medium

Tumor conditioned medium (CM) was harvested 72 hours after viral infection. Therefore, 150,000 tumor cells per well were plated in 6-well-plates, and infected at indicated MOI. Supernatant was collected, centrifuged (1,000 x g per minute, 10 minutes), used directly for co-culture experiments or stored at -80°C for downstream experimentation.

### 2.9 Multiplex cell death and IFN-g assay

In this work, the Cell Death 4-Plex Human ProcartaPlex™ Panel as well as Bio-Plex Pro Humane Cytokine IFN-g Singleplex was used according to manufacturer’s recommendations to characterize collected CM (see above). Sample acquisition was performed with a Bio-Plex 200 System (Luminex 200). Detection ranges of analytes are as follows: HMGB1 190 – 776200 pg/mL, HSP70 88 – 358900 pg/mL, HSP90 152 – 624500 pg/mL, IFN-g 2.3 - 20236 pg/mL.

### 2.10 T cell proliferation assay

The proliferation of CHM1^319^/HLA-A*02:01-restricted TCR-transgenic CD8^+^ T cells was measured by flow cytometry after T cell labelling with 10 M eFluor450 dye according to supplier’s recommendation (#65-0842-85, Thermo Fisher Scientific). T cells were co-cultured with CHM1^319^-peptide-pulsed THP-1 cells [4 hours with 10 g/mL peptide (Thermo Fisher Scientific) and 20 g/mL β_2_-microglobulinSigma Aldrich)], which were pre-cultured in CM for 72 hours, at a 2:1 ratio in T cell medium supplemented with 30 U/mL IL2, as described before (41). 75.000 THP-1 cells were seeded in 1 mL of CM in 24-well-plates. At start of T cell co-culture, THP-1 cells were counted, pulsed with peptide (-control) and T cells were added at a 2:1 ratio. 5 days after start of co-culture, T cell proliferation was analyzed in a MACSQuant Analyzer 10 after dead cell exclusion using DAPI or Viobility dye (both Miltenyi Biotech), also see **Supplementary Figure 4**. Furthermore, supernatant of one representative experiment was used to determine IFNγ-release.

### 2.11 Flow cytometry

All flow cytometric analyses were performed on a MACSQuant Analyzer 10. A single cell suspension with PBS was stained in 96-well-plates with primary fluorochrome-coupled antibodies. For REA antibodies, incubation time was 15 minutes at 4°C. Antibody concentrations for staining are provided as supplemental material. Single cell suspension from xenografts were used at a maximum of 2 x 10^6^ cells per well, using REA antibodies. Cells from *in vitro* experiments were used at a maximum of 2 x 10^6^ cells per well. Multicolor staining panels (*ex vivo*, *in vitro* DC maturation and T cell activation experiments) were auto-compensated according to supplier’s recommendation (MACSQuant Comp Bead Kit anti-REA). Dead cell exclusion was either done by DAPI, PI, or the fixable Viobility 405/520 dye). At the respective figure (legends) controls are indicated: i.e. fluorescence-minus one (FMO)- or isotype controls. Washing steps with were included before and after staining, after using fixable dye and before acquisition.

### 2.12 Flow cytometry data analysis and gating strategy

FCS raw data was processed using FlowJo V10. Gating strategy was applied in all analyses as follows: doublet discrimination in FSC-H versus FSC-A ➔ identification of cells in SSC-A versus FSC-A ➔ dead cell exclusion using DAPI (V1 vs. V2) PI (B3 vs. B2), or Viobility dye (V2 vs. V1 or B1). When several populations were present, specific cells were identified as follows: human T cells by double-positivity for CD45/CD3 (or CD3/CD4 or CD3/CD8), human tumor cells by mouse and humane double-negativity for CD45. If a specific marker expression in respective subsets was analyzed, isotype or FMO controls were applied (indicated in figure legends).

### 2.13 RT-qPCR

For explanted xenografted tumors, the RNeasy Mini Kit (Quiagen) was used according to manufacturer’s recommendation and RNA concentration was determined with the Nano Photometer Pearl (Implen). cDNA was produced using the High-Capacity cDNA Reverse Transcription Kit (Applied Biosystems) according to the supplier’s protocol. Reverse transcription was done using the iCycler (Bio-Rad Laboratories) with a 3-step-setup (1): initiation at 25°C for 10 minutes (2), elongation at 37°C for 120 minutes, and (3) inactivation at 85°C for 5 minutes. Human gene transcripts from explanted tumors were detected using 50 ng cDNA per reaction. Total reaction volume was adjusted in 96-well MicroAmp Fast Optical Reaction Plates was 15 μL, comprised of 7.5 μL GoTaq qPCR master mix, 0.75 μL of forward and reverse primers each, 1 μL of DEPC-H2O, and 5 μL of cDNA in DEPC-H2O. Reaction and recording of fluorescence were done in a StepOnePlus Real-Time PCR System (Applied Biosystems), according to manufacturer’s recommendation. To confirm specific PCR products, a melting curve analysis was performed. Applied primers are further described in the supplemental methods section. Relative quantification was done using the ΔΔCT-method. Beta-actin was used as housekeeping gene.

### 2.14 *In vivo* experiments

**Figure 2:**
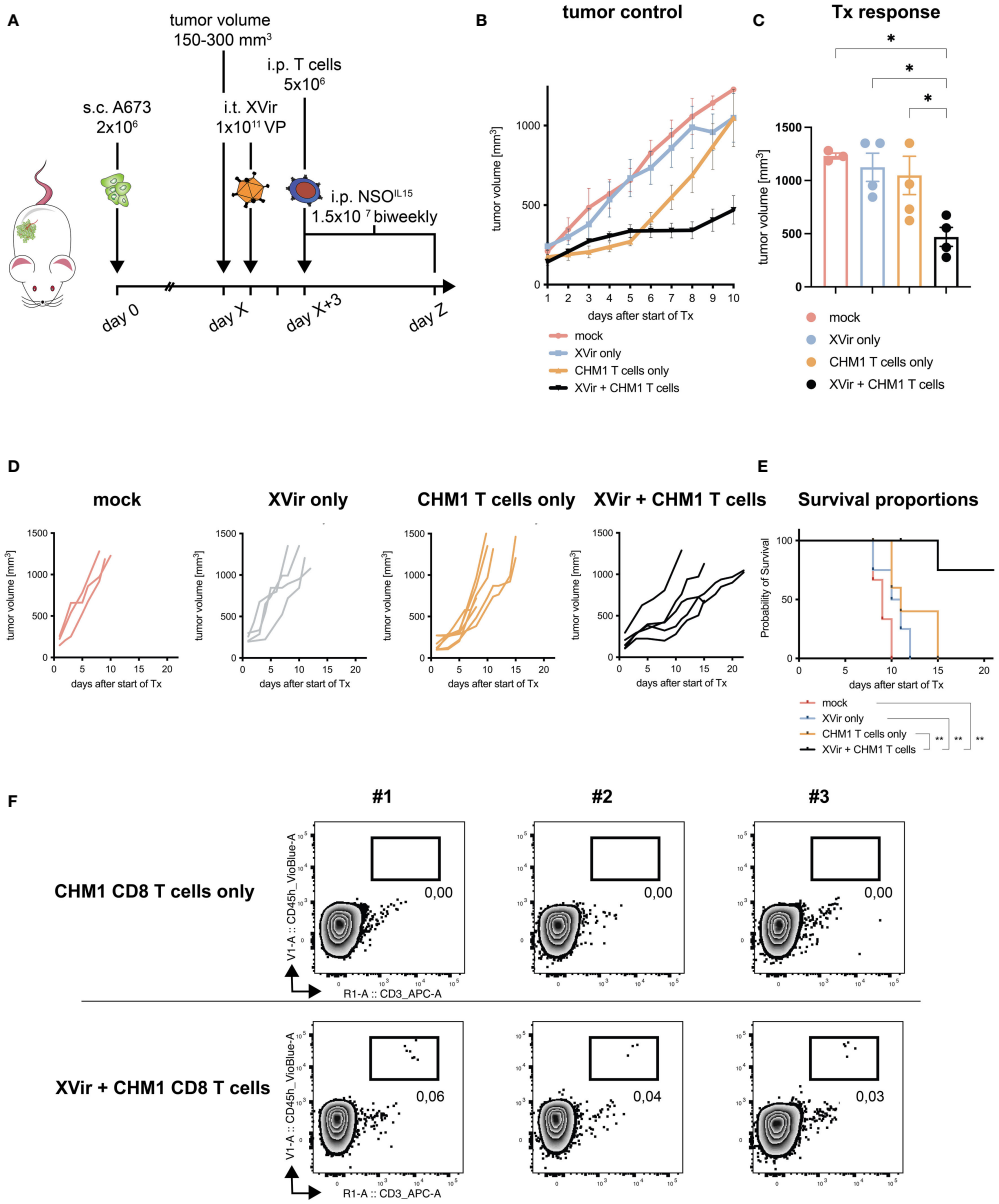
Combination of CHM1^319^/HLA-A*02:01-restricted TCR-transgenic CD8^+^ T cells (CHM1 CD8 T cells) and XVir-N-31 increases therapy response and survival. **(A)** Experimental setup: 2x10^6^ A673 tumor cells were injected s.c. in the flank of Rag2^-/-^c^-/-^ mice at day 0. When tumors reached a volume of 150-300 mm^3^ (day X), animals were randomized to treatment groups: *mock, Xvir (XVir-N-31) only, CHM1 (CD8) T cells only*, and *XVir + CHM1 T cells*. Then, 1x10^11^ viral particles (VP) of XVir-N-31 or PBS were injected i.t. at day X+1. 2 days later (day X+3), 5x10^6^ purified CHM1 T cells (validated by flow cytometry before administration, data not shown) were administered i.p. combined with 1.5x10^7^ IL-15-producing NSO cells i.p. (previously irradiated with 100 Gray). NSO application was repeated biweekly until the end of experiment (defined by tumor volume 1000 mm^3^). Tumor control in treatment groups is plotted as mean tumor growth until 10 days after start of therapy (Tx) in **(B)** and for each animal per group until the end of experiment in **(D)**; *mock* n=3, *XVir only* n=4, *CHM1 T cells only* n=5, and *XVir + CHM1 T cells* n=5. For B, imputed values were added to draw the tumor volume growth curve, assuming linear increase of tumor volume. **(C)** Response to Tx was analyzed on day 8-10 after start of Tx, using the maximum tumor volume per animal. Statistical analyses were performed in Prism 9 using the multiple comparison tool in combination with ordinary one-way ANOVA. **(E)** Kaplan-Meier survival curves were generated with Prism 9 from all animals the Mantel-Cox log-rank test. If animals did not reach the endpoint criteria tumor volume 1000 mm^3^, they were censored. **(F)** Percentage of tumor-infiltrating T cells (TILs, i.e. *CHM1 T cells*) in explanted tumors (in relation to all gated cells, after live-dead-cell exclusion with Viobility Dye) was evaluated at the end of experiment from 3 representative animals per group (*CHM1 T cells only* and *XVir + CHM1 T cells*) via flow cytometry using anti-human CD45-VioBlue and anti-human CD3-APC antibodies. Significance is indicated as asterisks: * p < 0.05, ** p < 0.01. Error bars indicate the SEM.

Animal experiments were approved by the local regulatory authorities in accordance with the German Federal Law and institutional guidelines (permission number of Regierung von Oberbayern: 55.2-2532-Vet_02-15-102). Rag2^-/-^ γc^-/-^ mice were bred and maintained in our animal facility center for Preclinical Research, School of Medicine and Health, TUM) under SPF conditions. Animals used for experimental and control conditions were bred and co-housed in the same rooms. The experiments were performed on 12-30-week-old male and female mice. A673 tumors were subcutaneously injected with 2 x 10^6^ cells in PBS per animal. Tumor size was determined every second day by caliper measurement und the tumor volume was calculated using the formula: volume = 0.5 x length x width^2^. Mice were randomly assigned to treatment/control groups when the tumor volume exceeded 150 – 300 mm^3^. Mice received intratumoral injections of 50 μL of XVir-N-31 (1 x 10^11^ viral particles, VP) or PBS. Two days afterwards, 5 x 10^6^ therapeutic T cells were injected intraperitoneally (i.p.) together with 1.5 x 10^7^ IL15-producing NSO cells (irradiated before with 100 Gy). NSO application was continued biweekly until the end of the experiment (tumor volume ≥ 1000 mm^3^, or other health-related reasons for experimental discontinuation as defined in the approval of the Regierung von Oberbayern). The experimental setup and timeline are given in **Figure 2A**.

### 2.15 Harvesting and processing of tumors and spleens

Mice were euthanized by isoflurane narcosis and cervical dislocation. Tumors and spleens were dissected with a scalpel as a whole in 3 mL PBS. Next, 1 mL of digestion buffer, consisting of RPMI1640, collagenase IV (200 U/mL, Sigma Aldrich), and DNase I (100 μg/mL, Thermo Fisher Scientific) was added. Enzymatic digestion was performed for 30 minutes at 37°C. Afterwards, samples were washed, filtered (70 μm) and acquired in standard medium for RNA isolation, flow cytometry or cryopreservation. All steps were performed on ice.

### 2.16 Computational analyses

**Figure 3:**
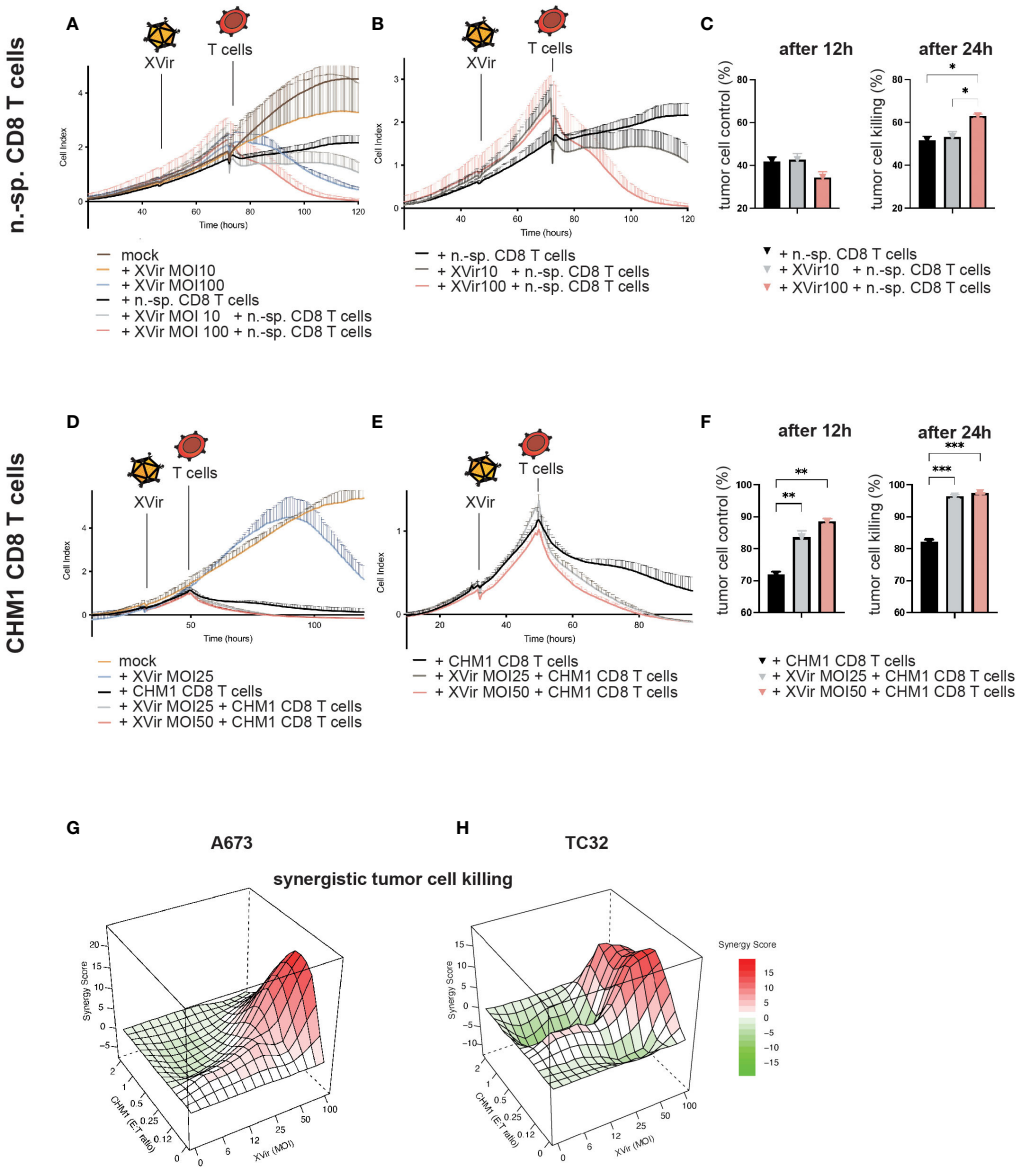
CHM1^319^/HLA-A*02:01-specific TCR-transgenic CD8^+^ T cells (CHM1 T cells) and XVir-N-31 synergistically lyse tumor cells *in vitro*. **(A)** Contact-dependent tumor growth of A673 was studied in xCELLigence assays to determine tumor cell detachment (i.e. killing) after XVir-N-31 at indicated MOI and n.-sp CD8 T cells (E:T = 1:1) and combinations of both, with respective controls. Infection with XVir-N-31 and addition of T cells is indicated with symbols. **(B)** Data complexity from **(A)** was reduced for better comprehensibility to experimental conditions only containing T cells. **(C)** T cell-mediated tumor cell killing was analyzed 12h and 24h after addition of T cells by subtracting respective cell index values of e.g. *mock* from *n.-sp. T cells*. **(D–F)** CHM1 CD8 T cell killing of A673 tumor cells was evaluated at indicated MOI, as described for n.-sp. CD8 T cells. **(G, H)** The probability of synergistic interactions using XVir-N-31 and CHM1 CD8 T cells with regard to tumor killing was assessed by SRB assays at 24h after addition of T cells (i.e. 48hpi) together with the *SynergyFinder tool* in R (experiments were performed in biological replicates and repeated, n=2). Synergy score explanation: interactions larger than 10 are considered as likely *synergistic*; interactions from -10 to 10 are most likely *additive* and interactions less than -10 are likely to be *antagonistic*. Significance levels are indicated as asterisks: * p < 0.05, ** p < 0.01, p*** < 0.005; Tukey’s multiple comparison within ordinary ANOVA was performed in **(C)** and **(F)** using Prism 9. Error bars indicate the SEM.

Prism 9 was used for statistical analyses. Student’s t-test was applied for normally distributed values. For more than 2-group comparison the one-way ANOVA was applied. Differences in animal survival were assessed by Mantel-Cox log-rank testing. Additional information for analysis of animal data is provided in **Figure 2**. For **Figure 1A**, the *Cell Line Selector*-tool from the DepMap Portal (Broad Institute: depmap.org) was used as described in respective figure legend. In **Figure 3**, the likelihood of synergistic interaction was analyzed using the Synergy Finder tool in R as recommended (42, 43). For input data, an excel file of biological replicates of experimental groups, controlled for mock-treatment, were used. Experiments were repeated at least twice. Data presented in **Supplementary Figure 1** was extracted from the R2 database (44).

## 3 Results

### 3.1 Ewing sarcoma cell lines are susceptible to adenovirus cell entry and XVir-N-31-infection induces tumor lysis and release of viral progeny

As a first step, EwS cell lines used in this project were examined for surface receptors associated with Ad species C type 5 cell entry, namely the natural *Coxsackie and Adenovirus Receptor* (CAR, CXADR) and additional receptors associated with adenovirus (Ad) entry equipped with the RGD-motif according to published reports consisting of *Integrin Subunit Alpha V* (ITGAV), *Integrin Subunit Beta 3* and *Beta 5* (ITGB3/ITGB5). Respective gene expression values (*Expression 21Q3 Public*) were extracted with the online-tool *Cell Line Selector* from the *DepMap Portal* of the *Cancer Cell Line Encyclopedia* (CCLE) for a selection of EwS and other pediatric sarcoma cell lines (**Figure 1A**). From available cell line data, EwS cell line A673 and CHLA9 showed the lowest expression of CAR. ITGAV expression was comparable, ITGB3 was close to absent and ITGB5 showed high variation in EwS cells. To get a better understanding about efficacy of XVir-N-31 virotherapy for downstream experiments, we examined CAR surface expression by FACS analysis on the 3 representative EwS cell lines A673, SK-N-MC, and TC32 used in later analyses. Interestingly, CAR surface expression correlated with relative gene expression analyses for A673 showing lowest expression, as compared to SK-N-MC with both higher CAR surface and relative expression levels (**Figures 1B, C**).

Furthermore, we exploited published transcriptome data from patient biopsies from the *R2 database* regarding aforementioned receptors associated with Ad cell entry (44). For EwS all 4 available data sets were examined, showing lower CAR expression than in other entities which were used for comparison (e.g glioblastoma or medulloblastoma). Of note, CAR expression was still higher than in healthy tissues *blood* and *muscle*, also included as controls. The expression of ITGAV, ITGB3, and ITGB5 in EwS was comparable to other tumor entities (**Supplementary Figure 1**).

Analyzing susceptibility of EwS for Ad infection, we infected a panel of cell lines with an E1A-deleted adenovirus carrying GFP as transgene and the RGD-motif for best comparability with XVir-N-31. Here, tested cell lines could all be infected but to a lesser degree than HEK293 cells (positive control), which are also used for Ad production (**Figure 1D**).

Next, we tested the oncolytic potential of XVir-N-31 in EwS cell lines using increasing multiplicities of infection (MOI) to assess dose-dependent effects. All cell lines were lysed at MOI 100 after 4-5 days post infection (dpi), including A673 which presented to be least sensitive to XVir-N-31-mediated oncolysis (**Figures 1E, F**). Of note, all analyzed cell lines supported viral replication resulting in novel viral progeny, assessed 48 hours post infection (hpi) and 72hpi by hexon-titer-tests (**Figure 1G**). As the replication of XVir-N-31 depends on any factors, including nuclear YB-1 expression and the capacity of E1A12S to activate viral expression, infectivity and oncolysis should be considered separately.

After establishing productive XVir-N-31-infection with consequent EwS cell lysis and *de novo* production of infectious particles, EwS-redirected CD8^+^ T cells retrovirally transduced to express an HLA-A*02:01-restricted TCR recognizing the nonameric peptide CHM1_319_ (*VIMPCSWWW*) derived from the source protein *Chondromodulin-1* were tested in combination with XVir-N-31.

### 3.2 CHM1^319^-specific TCR-transgenic CD8^+^ T cells (CHM1 CD8 T cells) and XVir-N-31 synergistically lyse EwS *in vitro*

First, contact-dependent tumor growth and detachment of tumor cells (i.e. cell killing) after mono- and combination therapy were studied using the xCELLigence assay. Therefore, T cell killing activity of non-specific (n.-sp.) purified CD8^+^ T cells at an *effector-to-target* (E:T) ratio of 1:1 with XVir-N-31 at MOI10 and MOI100 was examined initially. Infection of tumor cells was performed 24h before addition of T cells. Here, an increase of T cell-mediated killing was only observed with MOI100 (**Figures 3A–C**) indicating that peptide-HLA/TCR-independent mechanisms might be in place after infection with high MOI (45).

When using CHM1 CD8 T cells in combination with XVir-N-31, an increase of tumor cell killing was measured already 12h after addition of T cells with an increase of killing activity in a MOI/dose-dependent manner (**Figures 3D–F**).

For additional assessment of synergistic effects, tumor cell survival of cell lines A673 and TC32 were analyzed 48hpi and 24h after addition of CHM1 CD8 T cells using SRB assays and the R tool *SynergyFinder* (42, 43). Synergistic effects were predicted at MOI50-100 and E:T ratios of 1:1 to 0.25:1. Lower MOI were associated with mostly additive effects in these settings (**Figures 3G, H**; **Supplementary Figure 2**).

### 3.3 The combination of CHM1^319^/HLA-A*02:01-restricted TCR-transgenic CD8^+^ T cells (CHM1 CD8 T cells) and XVir-N-31 improves therapy response and survival in a preclinical mouse model

After the establishment of possible synergistic effects, when combining CHM1 T cells and XVir-N-31 *in vitro*, we moved to *in vivo* testing. Here, we used an adaption of our established xenograft mouse model, consisting of subcutaneously xenografted A673 tumors, with intratumoral administration of XVir-N-31 (XVir) and consecutive intraperitoneal application of therapeutic T cells (39, 41), as illustrated in **Figure 2A**.

We could demonstrate improved tumor control of the *combination* approach (*XVir + CHM1 T cells*) as compared to all monotherapies and controls when assessing therapy response at day 8-10 after start of treatment (**Figures 2B–D**). Furthermore, when taking the longitudinal tumor growth into account, we also could demonstrate a significant improvement in tumor control for the *combination* (**Table 2**). When comparing survival proportions, animals having received the *combination* exhibited a significant increase in median survival of 22 days as compared to 9 days for *mock*, 10.5 days for *XVir-N-31 only*, and 11 days for *CHM1 CD8 T cell only* (**Figure 2E**). Tumor-infiltration of human adoptively transferred T cells (TILs) was analyzed at the end of experiment. Here, TILs could only be detected in the experimental group of *XVir + CHM1 T cells*, albeit in very low frequencies (**Figure 2F**).

**Table 2:** Selected pairwise comparison for longitudinal tumor growth with Bonferroni adjustment using TumGrowth (46).

Grp compared to *combination*	Contrast	P value	P value adjusted
*mock*	91.64 [42.78; 140.50]	0.001	0.003
*XVir only*	73.84 [28.89; 118.79]	0.003	0.009
*CHM1 T cells only*	67.93 [25.49; 110.37]	0.004	0.011

Groups (Grp) were compared to the combination (XVir + CHM1 T cells). Contrast, p value and adjusted p value are indicated.

We did not detect significant differences between treatment groups *CHM1 T cells only* monotherapy and the *XVir + CHM1 T cells* combination with regard to antigen presentation (*Major Histocompatibility Complex*, MHC class I expression), expression of the ‘*don’t-eat-me*’-signal CD47, lymphocyte adhesion molecule ICAM-1 (*Intercellular Adhesion Molecule 1*), the T cell co-stimulatory molecule CD83, immune checkpoint molecules PD-1, PD-L1, or PD-L2 by flow cytometry staining (**Supplementary Figure 3A**). Also, transcriptomic data including HLA-A, HLA-B and CXCL10 expression did not differ (**Supplementary Figure 3B**). Only a tendency of improved T cell engraftment in analyzed spleens was observed for the combination therapy as compared to *CHM1 T cells only* (**Supplementary Figure 3C**). Of note, the number of analyzed animals and FACS staining without isotype controls constitutes a limitation of this conclusion.

### 3.4 XVir-N-31 induces immunogenic cell death markers and maturation of antigen-presenting cells, increasing CHM1^319^/HLA-A*02:01-restricted TCR-transgenic CD8^+^ T cell (CHM1 CD8 T cell) activity

**Figure 4:**
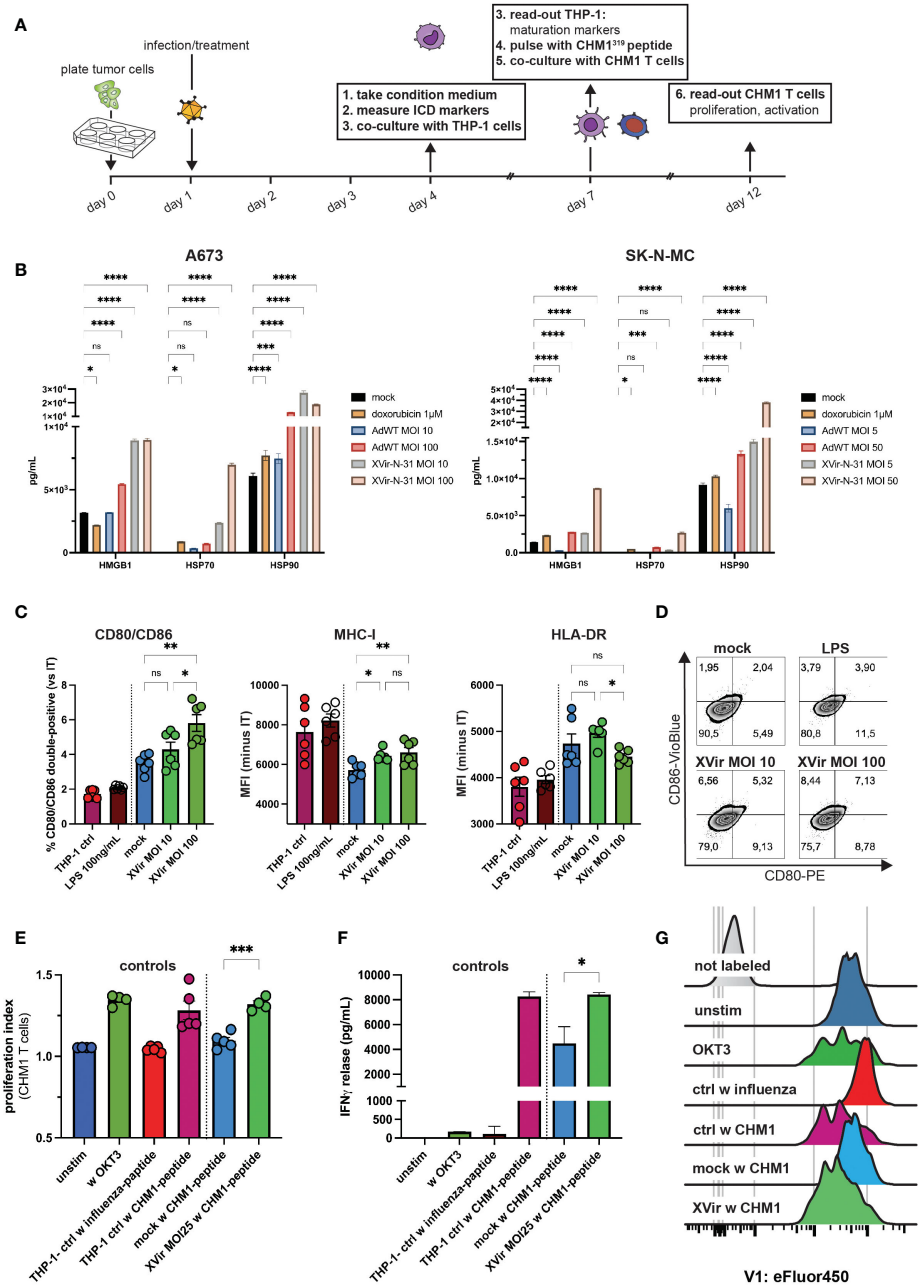
XVir-N-31 induces release of markers of an immunogenic cell death, upregulates maturation markers on antigen-presenting cells and stimulates antigen-specific T cell proliferation. **(A)** Experimental setup: tumor cells were plated in 6-well-plates. The day after, infection with XVir-N-31 or adenovirus wildtype (AdWT) was performed at indicated MOI or cells were treated with doxorubicin (1M). At day 4 (i.e. 72h post infection, hpi), tumor-conditioned medium (CM) was harvested and **(B)** markers of immunogenic cell death HMGB1, HSP70, and HSP90 were assessed via the luminex 200 system. Then, THP-1 cells were cultured in CM for 72h (until day 7) and maturation markers were assessed via flow cytometry **(C, D)**. Indicated MOI of XVir-N-31 was used in **(C)**. THP-1 controls are as described in **(E)**. **(E–G)** T cell proliferation and activation were analyzed 5 days after start of coculture (day 12) after T cell labelling with eFluor450. Control THP-1 cells were cultured in either standard medium or LPS was added to cells in standard medium (positive control). Furthermore, T cells were either not stimulated, or stimulated with OKT3 (50ng/mL). After dead cell exclusion with propidium iodine (PI), singlet exclusion, analysis of surface markers in **(C)** or proliferation (eFluor450) and was performed and release of IFNγ was detected. Antigen specific T cell gating strategy is provided in **Supplementary Figure 4**. Conditions included: unstim = unstimulated T cells only, w OKT3 = only OKT3 50 ng/mL as control, THP-1 ctrl w influenza/CHM1-peptide = THP-1 cell cultured in standard medium and pulsed with influenza-control or CHM1-peptide, mock and XVir MOI25 w CHM1-peptide. Statistical analysis was performed with Tukey’s multiple comparison combined with ordinary one-way ANOVA in **(C)** and for statistics in **(E, F)**, unpaired student’s t-test was used. Results of two independent experiments were pooled and shown in **(C, E)**. Here, each dot represents one biological replicate. Experiments in **(F)** were performed in triplicates. (.) Representative proliferation peaks of T cells are shown as histograms. Significance levels are indicated as asterisks: * p < 0.05, ** p < 0.01, p*** < 0.005, ****p < 0.0001, ns = not significant. Error bars indicate the SEM.

To elucidate further immunostimulatory components of XVir-N-31, we performed further *in vitro* analyses, focusing on markers of immunogenic cell death (ICD), maturation of antigen-presenting cells and consecutive abilities to stimulate antigen-specific T cell activity, using the experimental setup described in **Figure 4A**. First, markers of immunogenic cell death HMGB1, HSP70 and HSP90 were analyzed in the supernatant of XVir-N-31- and adenovirus wildtype (AdWT)-infected EwS cell lines A673 and SK-N-MC. Here, conditions of XVir-N-31 induced the highest release of ICD markers in mostly a dose-dependent manner for all analytes (compare **Figure 4B**).

When exposing THP-1 cells to respective tumor-conditioned media (i.e. A673 supernatants; CM), which represents a published model to study activation of antigen-presenting cells (41, 47, 48), analyses of surface markers after co-culture indicated increased activation and expression of the T cell-co-stimulatory receptor CD80 and CD86. Furthermore, decrease of MHC-I expression by CM from non-infected EwS cells (mock) was partly compensated by XVir-N-31 in this model (**Figure 4C**). Also, CD80/CD86 expression was induced by increasing MOI of XVir (**Figures 4C, D**).

As a last step, we utilized *CHM1 CD8 T cells* for further co-culture experiments with respective conditioned THP-1 cells after loading them with the cognate peptide CHM1^319^ and studied T cell proliferation. We could demonstrate as well an increase of antigen-specific T cell proliferation for all tested MOI of XVir (**Figures 4E, G**; **Supplementary Figure 4G**) and a significant higher release of IFNγ in XVir-versus mock-conditions (**Figure 4F**), complementing the results of T cell proliferation.

## 4 Discussion

Immunotherapeutic targeting of solid pediatric malignancies is challenging. EwS is considered a poorly-T cell-infiltrated tumor entity with high metastatic potential (8, 49). A recent report described a dynamic intrapersonal heterogeneity concerning the immune cells composition within the TME, depending on treatment-naïve or 1^st^/2^nd^ relapsed biopsy status (3). Hence, further immunological insight is urgently needed. Cellular immunotherapy approaches have to address a multitude of obstacles: physical barriers, low to absent MHC class I expression, immunosuppressive innate cells of myelomonocytic origin, cancer-associated fibroblast-like tumor cells, chemo- and cytokines as well non-classical MHCs in the TME preventing an impactful T cell attack (8, 50–54). They additionally have to include properties to revert immunosuppressive systemic factors, such as fibroblastic/myeloid-derived suppressor cells, chronic inflammation or tumor-derived extracellular vesicles hampering proper antigen presentation and activation of DC to enable formation of antitumor T cell immunity (1, 2, 55).

In short, all major interconnections within the cancer immunity cycle, which need to be intact for successful sarcoma rejection by the immune system, are defective in EwS (56–58). The inherent features of EwS cells exhibiting embryofetal and mesenchymal features, leading to this highly stress adaptive and metastatic tumor, may explain why monotherapies thus far were ineffective (9). Next to breaking physical and local barriers limiting T cell attacks, circumvention of peripheral immune tolerance is most likely key for eliciting a long-lasting anti-EwS immunity (59).

In this regard, analyses of outcomes from advanced EwS patients treated with HLA-matched versus -mismatched hematopoietic stem cell transplants did not show a clinically reproduced graft-versus-EwS effect (60), although additional donor lymphocyte infusions and other immunostimulatory combinations might have indeed the ability to induce antitumor immunity in a subset of pediatric sarcoma patients (61). Consequently, addressing more than one interconnection within the cancer immunity cycle, aiming to overcome the threshold of immune activation to break immune tolerance, is the next logical step. Therefore, we analyzed if YB-1-based virotherapy in combination with tumor-redirected TCR-transgenic T cells has the capacity to overcome some of the immunosuppressive factors, leading to an increase in T cell activity and direct T cell killing of tumor-redirected TCR-transgenic T cells. The results obtained in this study show that this is the case.

We herein propose a combination approach exploiting (a) the oncolytic adenovirus XVir-N-31 not only to mediate direct EwS cell lysis, next to the induction immunogenic cell death and reversal of lacking antigen presentation, but also to increase (b) activity and direct T cell killing of tumor-redirected TCR-transgenic T cells. Furthermore, we depict differences of T cell activation by comparing the OV XVir-N-31 with AdWT and could carve out the specific advantage of XVir-N-31 to increase tumor-redirected T cell activity in an antigen-dependent manner. We did not perform phenotyping of TCR-transgenic T cells before *in vivo* application, which could have been helpful to determine possible improvements for future work, such as the addition of further immunostimulatory transgenes in our therapeutic setting. Despite TILs were very low in the combination approach, they were still detectable compared to the *CHM1T cells only* group, indicating that the addition of XVir-N-31 can be used to induce TILs. In addition, the observation of enhanced human T cell engraftment in spleens, suggests supportive effects of XVir-N-31 to increase *in vivo* persistence of adoptively transferred TCR-transgenic T cells.

We showed before that the combination of XVir-N-31 and CDK4/6 inhibition (CDK4/6i) has the capacity to jointly activate innate and adaptive immunity in EwS with superior tumor control and signs of an abscopal effect (41). Other recent work provides *in vitro* evidence that therapy with XVir-N-31 can increase the phagocytic potential of macrophages and DCs, which can be further enhanced by CD47 blockade (von Ofen et al., 2023 *in revision*). Colleagues also implementing XVir-N-31 and a derivate with a PD-L1 transgene, again observed signs of an abscopal effect in a humanized glioblastoma mouse model associated with the induction of ICD *in vivo* (62).

For our established tumor-redirected TCR-transgenic T cells, we now provide with XVir-N-31 an additional tool, to increase the efficacy of tumor-redirected T cells, as demonstrated here for *CHM1 T cells in vitro* and *in vivo.*

Concerning the high variability of CAR (CXADR) expression in EwS patient samples, the introduction of the RGD motif into the viral genome broadens the receptor repertoire for Ad entry, although virus-resistant tumor clones are a common phenomenon in clinical reality, reflected by the relatively high rate of non-responders in OV monotherapy approaches (27, 28). Nevertheless, OV not only targets (i) tumor cells directly but also (ii) the TME by triggering inflammatory responses, e.g. release of interferons in myeloid cells, as well as the (iii) tumor stroma, inducing the abrogation of immune cell-excluding matrix structures and tumor-promoting vasculature (63, 64). This emphasizes the simultaneous need to (a) increase viral replication through combination approaches, aiming at maximizing the antiviral/antitumor immune response, and to (b) select viral constructs with the inherent capability to trigger tumor-rejecting inflammatory pathways. Our results show that the CM of XVir-N-31-infected tumor cells efficiently render antigen presenting THP-1 cells into a state of superior T cell-stimulatory capacity, compared to AdWT, which actually is more potent in tumor cell killing than XVir-N-31 (65). This strongly indicates that not the superior cell killing of AdWT but rather the induction of an ICD by XVir-N-31 is playing a decisive role for this effect.

Regarding the reversion of an immunosuppressive TME as well as increased cell killing capacity by CDK4/6i and XVir-N-31, as shown by us, combined with recently published results on preconditioning of mouse or human CAR T cells with CDK4/6i and thereby enhancing their persistence and antitumor efficacy in murine models, we are particularly interested in including CDK4/6i in this combination approach (29, 41, 66). Prospectively, XVir-N-31 equipped with an anti-PD-L1 will be implemented as well, very likely minimizing toxicities observed in clinical trials using systemic application of ICB.

When studying oncolytic adenoviruses and therapeutic T cells in preclinical mouse models for translational purposes, one has to be aware of the inherent limitation. The herein applied xenograft model with transient humanization is lacking the crosstalk of innate and adaptive immunity. But as oncolytic Ads, as therapeutic products, only possess the characteristic to properly replicate in human cancer cells, and tumor-redirected T cell therapy for EwS has not yet been established in immunocompetent animal models, our approach addresses best the current need to develop immunotherapeutic combination strategies for EwS patients. A BMBF-funded phase-I clinical trial for pediatric sarcoma patients is in preparation using XVir-N-31, the CDK4/6i ribociclib and ICB with pembrolizumab (XVIR-RIB-PEM, 01EN2009). In case good tolerability and signs of antitumor immune activity is observed, the addition of tumor-redirected T cells boosting the adaptive arm of the immune system would be a highly attractive addition.

## Data availability statement

The original contributions presented in the study are included in the article/**Supplementary Materials**, further inquiries can be directed to the corresponding author/s.

## Ethics statement

The animal study was approved by Regierung von Oberbayern: 55.2-2532-Vet_02-15-102. The study was conducted in accordance with the local legislation and institutional requirements.

## Author contributions

SS: Conceptualization, Data curation, Formal Analysis, Funding acquisition, Investigation, Methodology, Project administration, Supervision, Visualization, Writing – original draft, Writing – review & editing. MT: Formal Analysis, Methodology, Writing – review & editing. HG: Investigation, Methodology, Writing – review & editing. AV: Investigation, Methodology, Writing – review & editing. PK: Formal Analysis, Methodology, Writing – review & editing. JE: Formal Analysis, Methodology, Writing – review & editing. CP: Formal Analysis, Investigation, Methodology, Writing – review & editing. CK-W: Methodology, Writing – review & editing. JH: Funding acquisition, Methodology, Writing – review & editing. SB: Conceptualization, Funding acquisition, Project administration, Supervision, Writing – review & editing. PH: Conceptualization, Investigation, Methodology, Project administration, Supervision, Writing – original draft, Writing – review & editing. UT: Conceptualization, Funding acquisition, Investigation, Methodology, Project administration, Supervision, Writing – original draft, Writing – review & editing.
