# Supplementary material for: TCR-transgenic T cells and YB-1-based oncolytic virotherapy improve survival in a preclinical Ewing sarcoma xenograft mouse model
Source: Front Immunol. 2024 Jan 22;15:1330868. doi: 10.3389/fimmu.2024.1330868 (PMC10839048; doi:10.3389/fimmu.2024.1330868)
Supplement: Supplementary file 1 [file DataSheet_1.pdf]

Schober SJ et al. (2023):

TCR-transgenic T cells and YB-1 based oncolytic virotherapy improve survival in a preclinical Ewing sarcoma xenograft mouse model

Supplementary Material

1. Primer sequences used in this study

Name	Specificity	Forward 5'-3'	Reverse 5'-3'
CXCL10	human	GTG GCA TTC AAG GAG TAC CT	TGA TGG CCT TCG ATT CTG GA
GAPDH	human	CTC TGC TCC TCC TGT TCG AC	ACG ACC AAA TCC GTT GAC TC
HLA-A	human	TCA CCC TGA GAT GGG AGC	ATG TGG AGG AGG AAG AGC T
HLA-B	human	CAG TTC GTG AGG TTC GAC AG	CAG CCG TAC ATG CTC TGG A
β actin	human	TAA GTA GGT GCA CAG TAG GTC TGA	AAA GTG CAA AGA ACA CGG CTA AG

2. Antibodies used in this study

2.1 Antibodies for hexon-titer-test (HTT)

Specificity	REF	Concentration	Method	Company
Adenovirus (hexon)	AB1056	1:1000	HTT	Merck Millipore
Rabbit anti-goat IgG-HRP	P0449	1:1000	HTT	Dako

2.2 Antibodies for flow cytometry (conjugated) – anti-human

Specificity	Fluorochrome	Clone	Concentration	Company
CD3	PE-Vio770	REA613	1:100	Miltenyi Biotech
CD4	APC-Vio770	REA623	1:100	Miltenyi Biotech
CD8	FITC	REA734	1:100	Miltenyi Biotech
CD25	APC	REA579	1:100	Miltenyi Biotech
CD40	VioBright FITC	REA733	1:100	Miltenyi Biotech
CD45	VioGreen	REA747	1:100	Miltenyi Biotech
CD45	APC	REA747	1:100	Miltenyi Biotech
CD47	FITC	REA220	1:100	Miltenyi Biotech
CD80	PE	REA661	1:100	Miltenyi Biotech
CD86	VioBlue	REA968	1:100	Miltenyi Biotech
CHM1-multimer	PE	-	1:100	Busch Lab
HLA-ABC	APC	REA230	1:100	Miltenyi Biotech
HLA-ABC	PerCP-Vio700	REA230	1:100	Miltenyi Biotech
HLA-DR	APC	REA804	1:100	Miltenyi Biotech
HLA-DR	PE-Vio770	REA805	1:100	Miltenyi Biotech
CD274 (PD-L1)	APC	REA1197	1:100	Miltenyi Biotech
CD274 (PD-L1)	VioBright B515	REA1197	1:100	Miltenyi Biotech
CD279 (PD-1)	APC	PD1.3.1.3	1:100	Miltenyi Biotech
REA Ctrl	APC	REA293	1:100	Miltenyi Biotech
REA Ctrl	FITC	REA293	1:100	Miltenyi Biotech
REA Ctrl	VioBright B515	REA293	1:100	Miltenyi Biotech

2.3 Further dyes and solutions used for flow cytometry

Application	Dye/Name	REF	Concentration	Company
Cell proliferation	eFluor450	65-0842-85	According to protocol	Thermo Fisher Scientific
Dead cell exclusion	DAPI staining solution	130-111-570	1:100	Miltenyi Biotech
Dead cell exclusion	Propidium iodide (PI = solution)	130-093-233	1:100	Miltenyi Biotech
Dead cell exclusion	Viobility 405/520 Fixable Dye	130-109-814	According to protocol	Miltenyi Biotech
