# Supplementary figures and images for: TCR-transgenic T cells and YB-1-based oncolytic virotherapy improve survival in a preclinical Ewing sarcoma xenograft mouse model
Source: Front Immunol. 2024 Jan 22;15:1330868. doi: 10.3389/fimmu.2024.1330868 (PMC10839048; doi:10.3389/fimmu.2024.1330868)

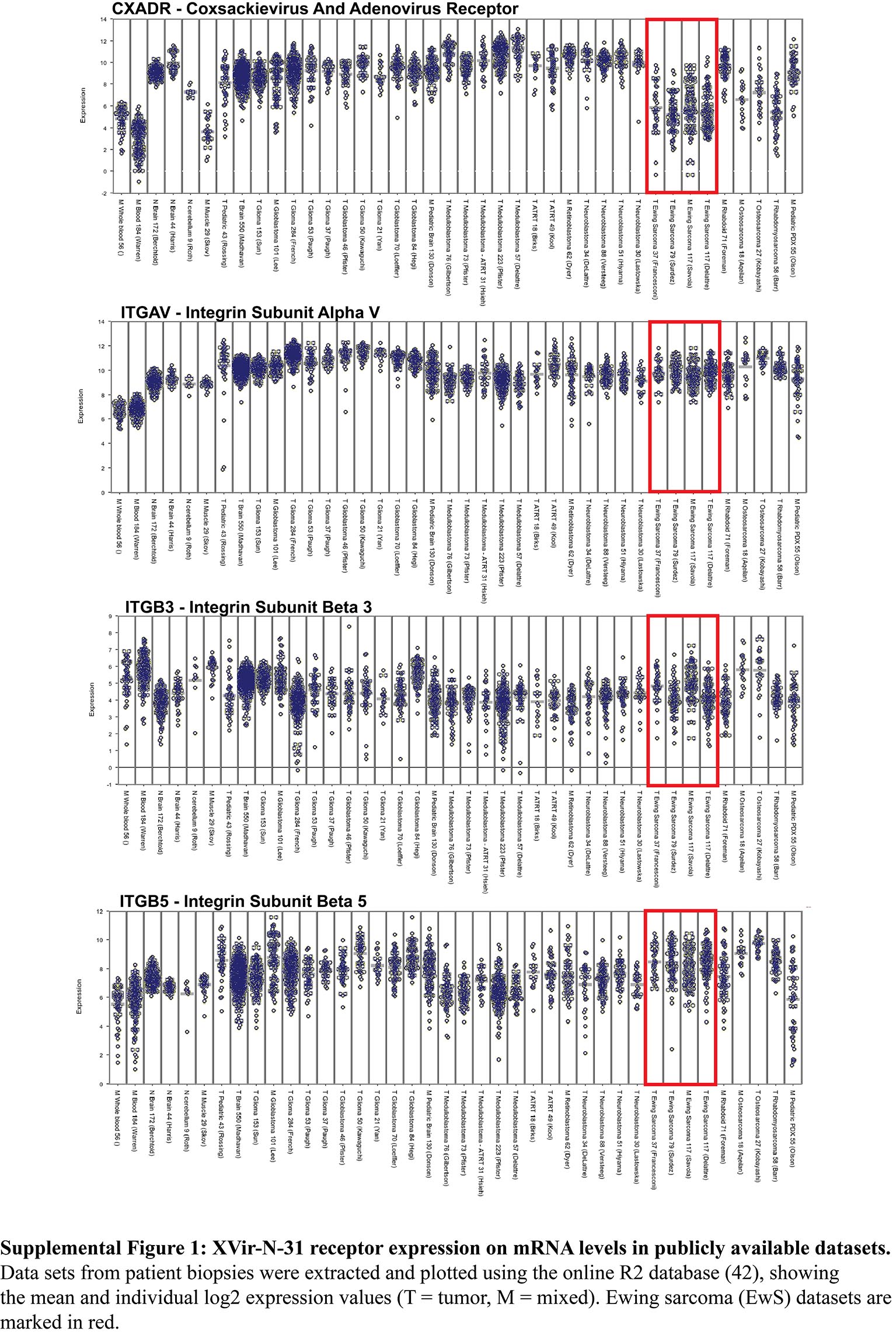

Supplement: Supplementary file 2 [file Image_1.jpeg]

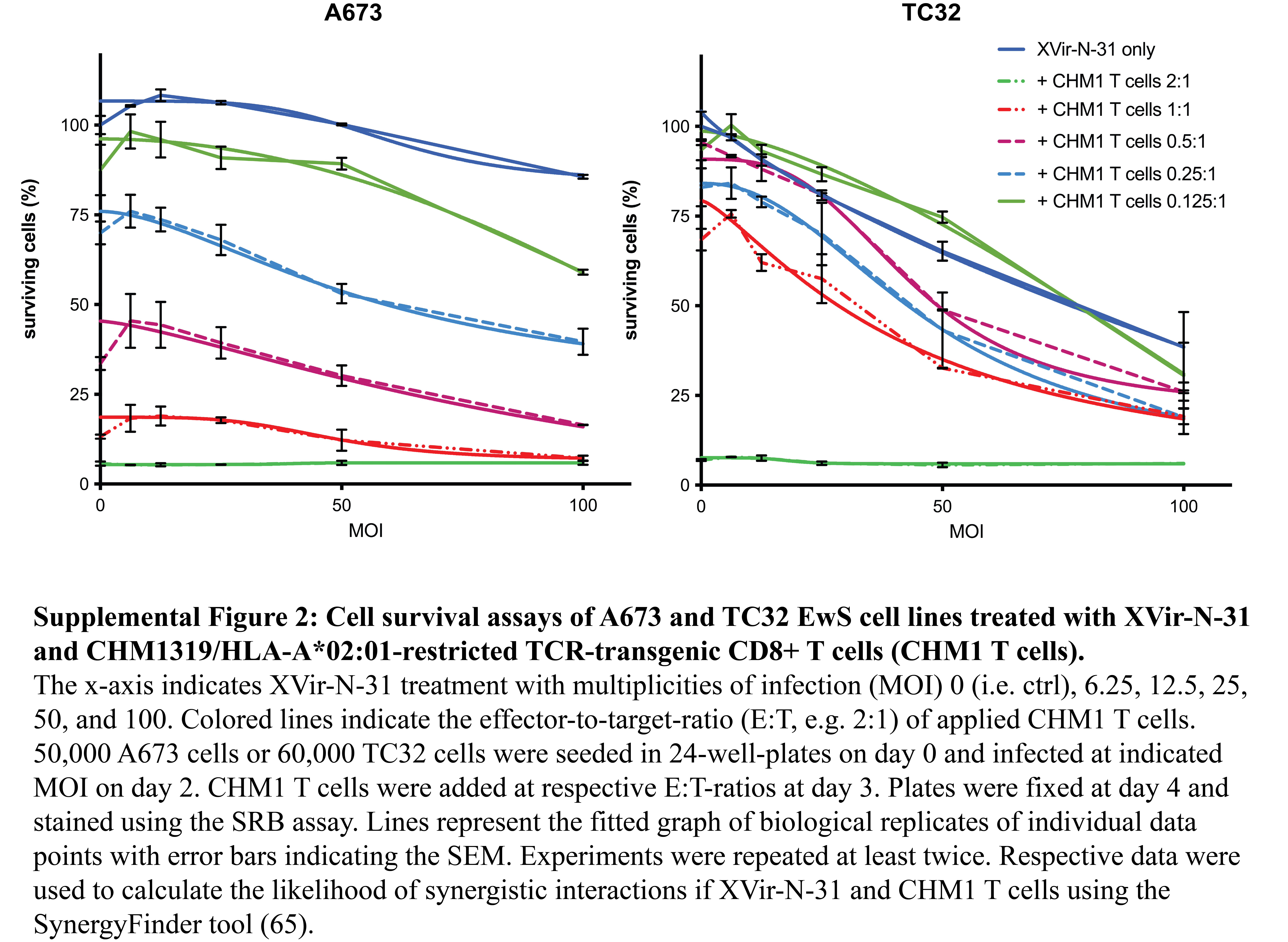

Supplement: Supplementary file 3 [file Image_2.jpeg]

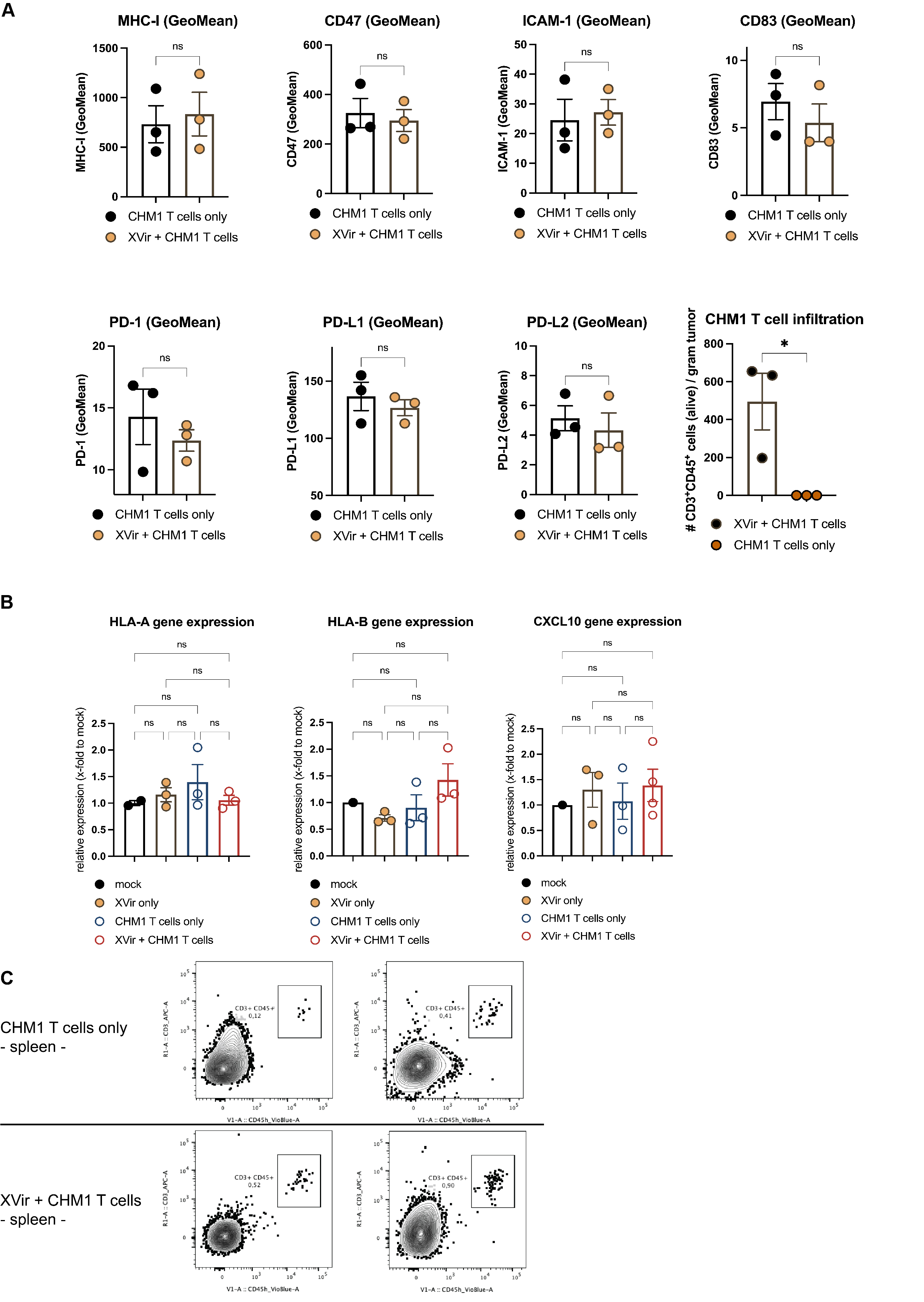

Supplement: Supplementary file 4 [file Image_3.jpeg]

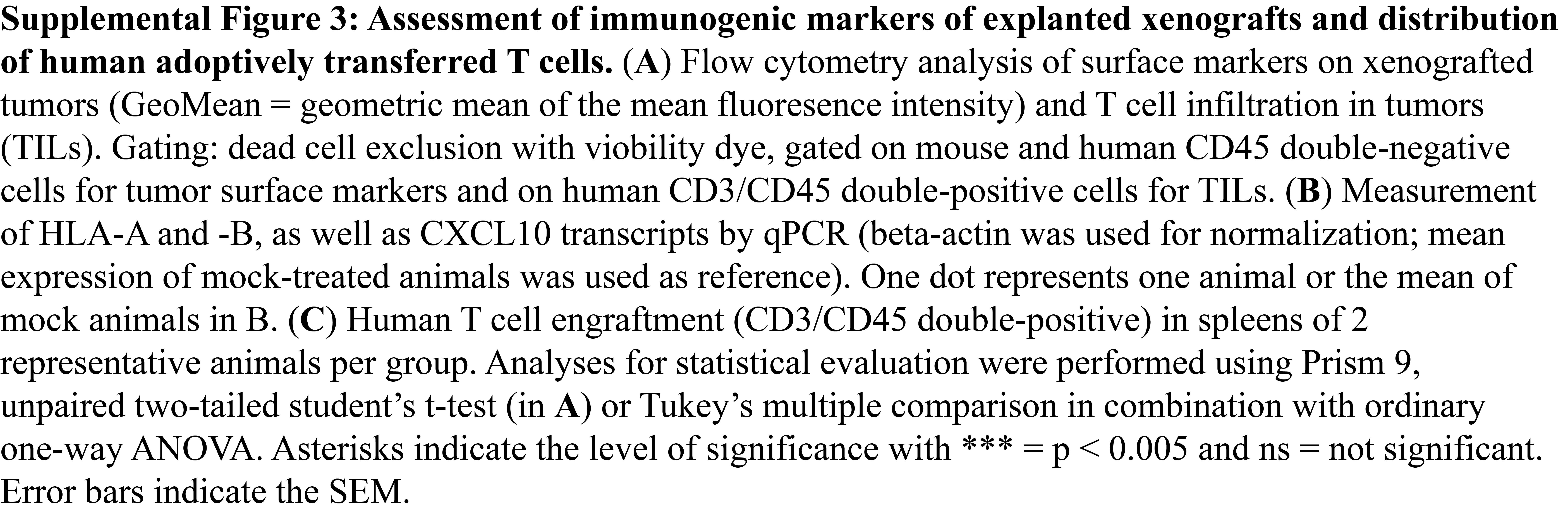

Supplement: Supplementary file 5 [file Image_4.png]

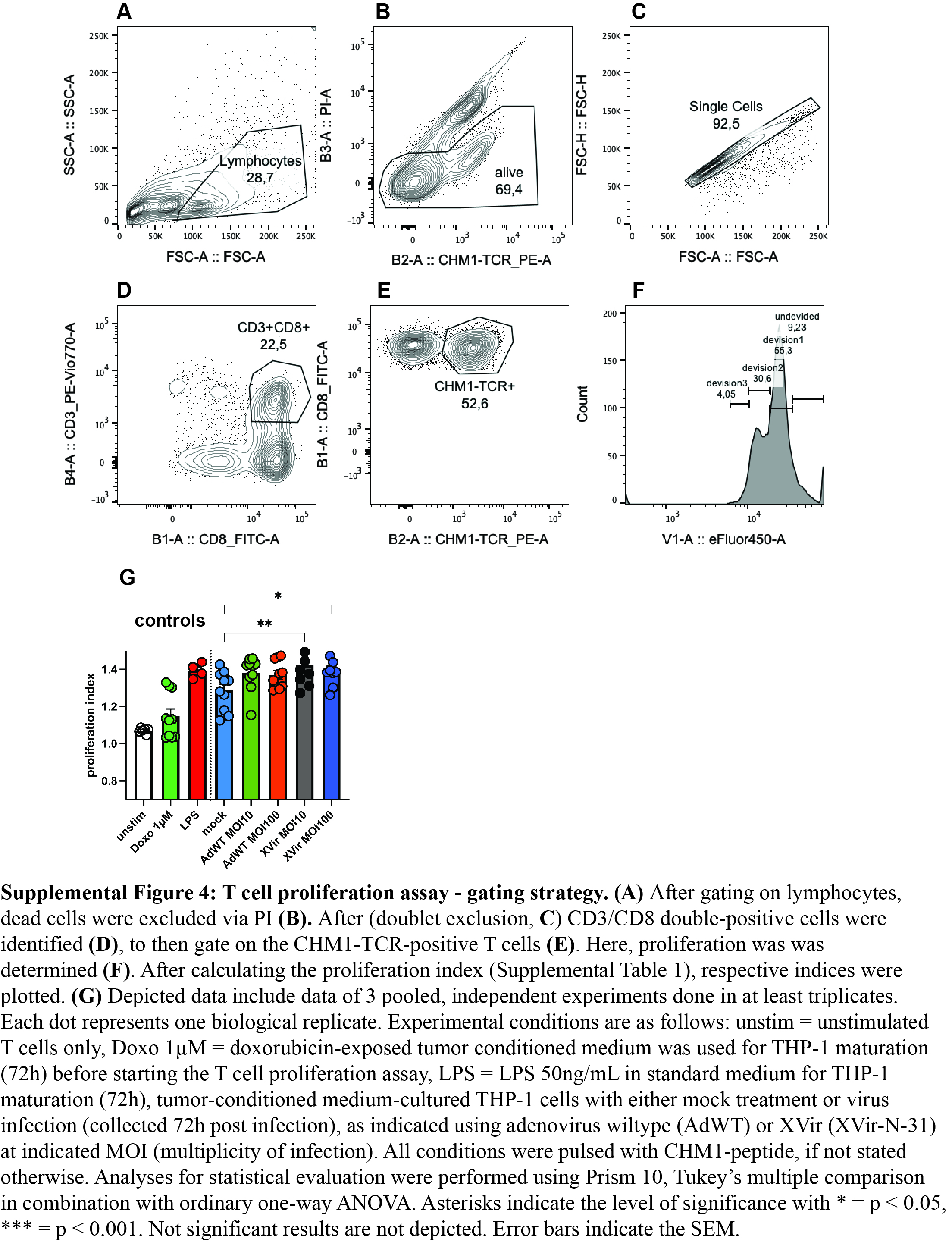

Supplement: Supplementary file 6 [file Image_5.jpeg]
